# P-1944. Previously Unrecognized Candida auris Gut Colonization in Critically Ill Patients

**DOI:** 10.1093/ofid/ofaf695.2112

**Published:** 2026-01-11

**Authors:** Luisannys I Lozada Pinto, Truc Cecilia Tran, Rodrigo P Baptista, Diana Panesso-botero, Marissa Schettino Intriago, Husna Malikzad, Shiva Murali, Andrea M Detranaltes, An Dinh, Kavindra Singh, Shubhra Singh, Luis Bejar, Asmita Ghosh, Roberta Higgins, Giselle Ortiz, Abigail A Amaya, Muhammad Virk, Kirsten Rydell, Mary North Jones, Rachel Atterstrom, Blake M Hanson, Samuel A Shelburne, Tor Savidge, David B Corry, J Christian Perez, Cesar A Arias, Max W Adelman

**Affiliations:** Houston Methodist Academic Institute, Houston, TX; Houston Methodist Research Institute, Houston, TX; Houston Methodist Research Institute, Houston, TX; Houston Methodist Hospital, Houston, TX; Houston Methodist Research Institute, Houston, TX; Houston Methodist Hospital, Houston, TX; Houston Methodist Research Institute and Weill Cornell Medical College, Houston, Texas; Houston Methodist Hospital, Houston, TX; Houston Methodist Research Institute, Houston, TX; Houston methodist research institute, Houston, Texas; Houston Methodist Hospital, Houston, TX; Houston Methodist Academic Institute, Houston, TX; Houston Methodist Hospital, Houston, TX; Houston Methodist Hospital, Houston, TX; Houston Methodist Hospital, Houston, TX; Houston Methodist Hospital, Houston, TX; Houston Methodist The Woodlands Hospitals, conroe, Texas; Houston Methodist Hospital, Houston, TX; Houston Methodist Research Institute, Houston, TX; Houston Methodist Hospital, Houston, TX; The University of Texas Health Science Center, Houston, Texas; University of Texas MD Anderson Cancer Center, Houston, Texas; Baylor College of Medicine, Houston, Texas; Baylor College of Medicine, Houston, Texas; UTHealth Houston, Houston, Texas; Houston Methodist and Weill Cornell Medical College, Houston, TX; Houston Methodist Hospital, Houston, TX

## Abstract

**Background:**

*Candida auris* is a drug-resistant fungus that poses a growing risk in healthcare environments, particularly among intensive care unit (ICU) patients. While other *Candida* species routinely colonize the gut, it is unknown if the gastrointestinal (GI) tract is a niche for *C. auris*. We aimed to determine the presence of *C. auris* in the gut of ICU patients and evaluate whether these isolates exhibit phenotypic traits that support intestinal colonization.

**Figure 1.**
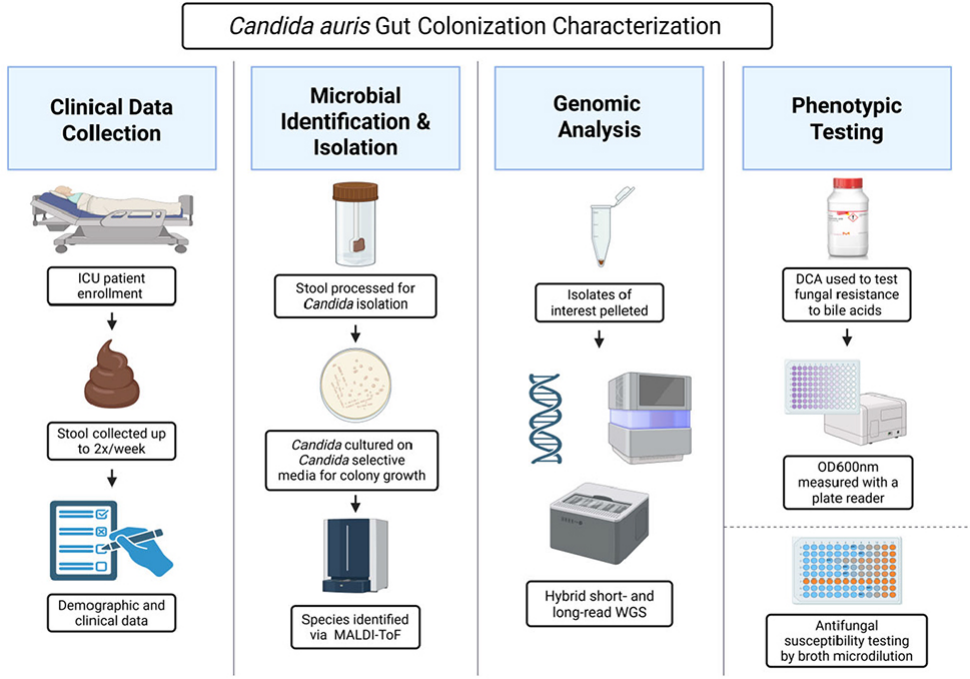
Abbreviations: DCA, deoxycholic acid; ICU, intensive care unit; MALDI-ToF, matrix-assisted laser desorption/ionization-time of flight; WGS, whole genome sequencing.

**Figure 2.**
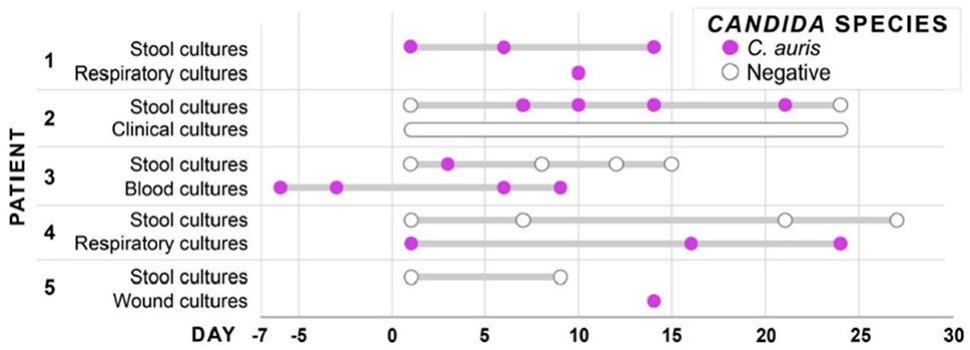
C. auris gut colonization and clinical detection in intensive care unit (ICU) patients.

**Methods:**

We assessed *C. auris* gut colonization in ICU patients by collecting stool samples and demographic/clinical information from enrolled patients from 01/2021-06/2024 (Figure 1). Stool samples were cultured on *Candida* selective media, isolates were identified using MALDI-ToF and their genomes were sequenced. To evaluate bile acid resistance, representative stool isolates of *C. auris* and *C. albicans* were exposed to increasing concentrations of the secondary bile acid deoxycholic acid (DCA), and growth was measured over time. Antifungal minimum inhibitory concentrations (MICs) were determined by broth microdilution.

**Figure 3.**
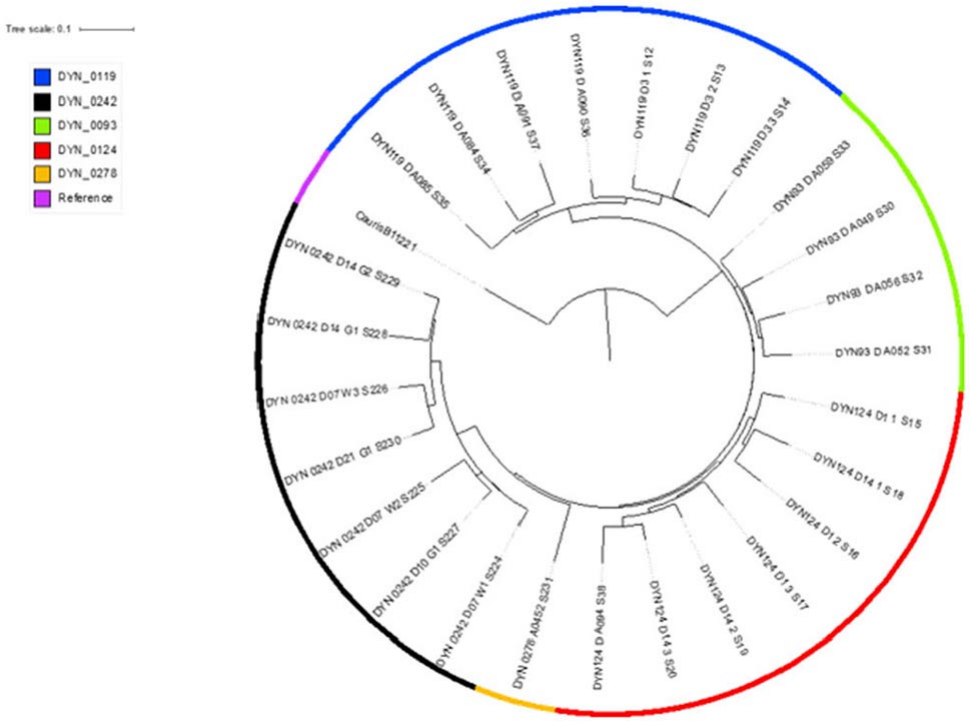
Phylogenetic tree of C. auris isolates (Clade III), collected in triplicate from each patient on different days from stool and clinical cultures.

**Figure 4.**
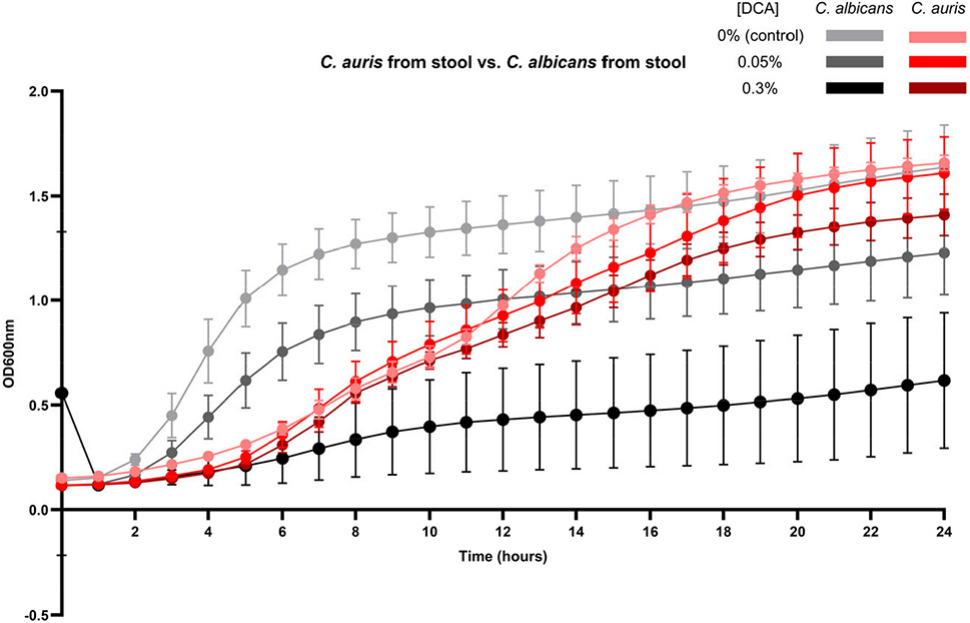
Growth of C. auris vs. C. albicans gut colonizing isolates in deoxycholic acid (DCA).

**Results:**

Of 150 ICU patients enrolled, the mean age was 60.8 ± 15.6 years, 83 (56%) were male, and 24% were Hispanic/Latino. Mean Charlson Comorbidity Index was 4.9 ± 3.0; 34 patients (23%) were solid organ transplant recipients, and 54 (36%) were in shock on ICU admission. *C. auris* was detected in stool cultures of three patients (2%), of which two had colonization at other body sites (Figure 2). Genomic analysis showed that these isolates were highly clonal and belonged to Clade III (Figure 3). All *C. auris* isolates were resistant to fluconazole and susceptible to micafungin. When exposed to DCA, *C. auris* stool isolates exhibited significantly less growth inhibition compared to *C. albicans* stool isolates. At high (0.3%) DCA concentrations, the mean inhibition rates were 17*%* for *C. auris* stool isolates and 66% for *C. albicans* stool isolates (Figure 4).

**Conclusion:**

These findings provide evidence of *C. auris* GI colonization in ICU patients. Moreover, the demonstrated resistance of *C. auris* stool isolates to DCA highlights its potential adaptive mechanisms for survival in the gut. These findings indicate that the GI tract may be a persistent reservoir for *C. auris* with implications for transmission and infection control.

**Disclosures:**

All Authors: No reported disclosures

